# Nanoporous Sodium Carboxymethyl Cellulose-*g*-poly (Sodium Acrylate)/FeCl_3_ Hydrogel Beads: Synthesis and Characterization

**DOI:** 10.3390/gels6040049

**Published:** 2020-12-11

**Authors:** Bijender Kumar, Ruchir Priyadarshi, Sauraj, Farha Deeba, Anurag Kulshreshtha, Kirtiraj K. Gaikwad, Jaehwan Kim, Anuj Kumar, Yuvraj Singh Negi

**Affiliations:** 1 Department of Polymer and Process Engineering, Indian Institute of Technology Roorkee, Roorkee 247667, India; bijenderkumarchem@gmail.com (B.K.); farha.deeba86@gmail.com (F.D.); 2 Creative Research Center for Nanocellulose Future Composites, Department of Mechanical Engineering, Inha University, Inharo, Michuhol-gu, Incheon 22212, Korea; jaehwan@inha.ac.kr; 3 Department of Food and Nutrition, Bio Nanocomposite Research Center, Kyung Hee University, Seoul 02447, Korea; ruchirpriyadarshi@gmail.com; 4 Department of Materials Engineering, Indian Institute of Science, Bangalore 560012, India; saurajpolymeriitd@gmail.com; 5 Department of Paper Technology, Indian Institute of Technology Roorkee, Roorkee 247667, India; anuragk77@gmail.com (A.K.); gaikwad.msu@gmail.com (K.K.G.); 6 School of Chemical Engineering, Yeungnam University, Gyeongsan 38541, Korea

**Keywords:** sodium carboxymethyl cellulose, poly(sodium acrylate), FeCl_3_, cross-linking, nanoporous beads, hydrogel, thermal analysis

## Abstract

Novel sodium carboxymethyl cellulose-*g*-poly (sodium acrylate)/Ferric chloride (CMC-*g*-PNaA/FeCl_3_) nanoporous hydrogel beads were prepared based on the ionic cross-linking between CMC-*g*-PNaA and FeCl_3_. The structure of CMC and CMC-*g*-PNaA were elucidated by Fourier transform infrared spectroscopy (FTIR) and nuclear magnetic resonance (NMR) spectroscopy, and the elemental composition was analyzed by energy dispersive X-ray analysis (EDX). The physicochemical properties of the CMC-*g*-PNaA/FeCl_3_ hydrogel beads were analyzed by X-ray diffraction (XRD), scanning electron microscopy (SEM), atomic force microscopy (AFM) and thermogravimetric analysis (TGA). The swelling percentage of hydrogel beads was studied at different time periods. The obtained CMC-*g*-PNaA/FeCl_3_ hydrogel beads exhibited a higher nanoporous morphology than those of CMC-*g*-PNaA and CMC beads. Furthermore, an AFM image of the CMC-*g*-PNaA/FeCl_3_ beads shows granule type topology. Compared to the CMC-*g*-PNaA (189 °C), CMC-*g*-PNaA/FeCl_3_ hydrogel beads exhibited improvement in thermal stability (199 °C). Furthermore, CMC-*g*-PNaA/FeCl_3_ hydrogel beads depicted a higher swelling percentage capacity of around 1452%, as compared to CMC-*g*-PNaA (1096%). Moreover, this strategy with preliminary results could be useful for the development of polysaccharide-based hybrid hydrogel beads for various potential applications.

## 1. Introduction

Hydrogels are three-dimensional polymeric network structures with a higher capacity to absorb water due to the presence of hydrophilic groups, but which are insoluble in water. Hydrogels are gaining significant attention of the researchers due to their application in various research fields, such as wastewater treatment, agriculture, tissue engineering, drug delivery and electroactive applications [1,2,3,4]. Currently, researchers have focused on the development of polysaccharide-based hydrogels due to their cost effectiveness, biodegradability and higher ability to remove pollutants as compared to synthetic polymeric hydrogels [5,6,7]. As polysaccharide, sodium carboxymethyl cellulose (CMC) is a high-molecular weight anionic polymeric material that contains numerous hydroxyl (–OH) and carboxylate (–COO^–^) groups [8]. Due to its biodegradability, biocompatibility and non-toxicity, it has been extensively used in tissue engineering, drug delivery, textile industry, paper, dispersant and water treatment applications [1,9,10,11].

Thus far, few polysaccharide-based hydrogel beads including alginate [12], CMC/dextran sulfate [13], calcium alginate-CMC [14], CMC-NPS [15], sodium alginate-CMC [16], alginate/Ca^2+^ [17], silsesquioxane/CMC [8] and cellulose nanocrystal/sodium alginate [18] have been developed with improved properties. In addition, Yanshu Shi et al. have reported the porous organic polymeric network and used it for the photothermal therapy, which was prepared by the metalation of polymers with Fe^3+^ ions [19,20]. Yang and co-workers reported CMC hydrogel beads that were prepared by the cross-linking of epichlorohydrin with CMC. The hydrogel beads showed the capability to adsorb toxic metals [21]. Agarwal et al. developed CMC-based hydrogel beads by ionic-gelation methods for drug delivery systems [14]. Similarly, Ren and co-workers developed the CMC gel-based beads via blending, followed by cross-linking, and also studied the effect of pH, temperature and dosage of beads on the adsorption behavior of Pb^2+^ [16]. Furthermore, Akalin and Pulat have reported cross-linked CMC hydrogel beads prepared by cross-linking between CMC chains with ferric chloride (FeCl_3_) as a cross-linker, and investigated the pore size, swelling and degradation behavior [22]. Recently, CMC/zinc oxide (ZnO)-based nanocomposite hydrogel was developed by the ionic cross-linking of CMC with Zn^2+^ ions. The cross-linked nanocomposite hydrogel showed high swelling properties and strong antimicrobial activity, which could be used in biomedical and superabsorbent applications [23].

In recent years, several studies have been undertaken in hydrogel materials of acrylic acid (AA)-grafted polysaccharides, namely CMC-*g*-PAA [24], chitosan/PAA [25], PAA-hydroxyethyl cellulose [26], Amylose-*g*-PAA [27] and various AA-grafted polysaccharides [28,29] for superabsorbent, waste water treatment and biomedical applications. Until now, ionically cross-linked nanoporous hydrogel beads of CMC-*g*-poly (sodium acrylate) (PNaA) have not been reported. In this work, we introduced the novel nanoporous CMC-*g*-PNaA/FeCl_3_ hydrogel beads that were developed by the ionic cross-linking of CMC-*g*-PNaA in the presence of FeCl_3_ as an ionic cross-linker, while CMC-*g*-PNaA was synthesized by the grafting of AA onto CMC in the presence of potassium persulfate initiator. In this study, the effect of ionic linkage on the thermal stability, pore size and swelling behavior of CMC-*g*-PNaA/FeCl_3_ hydrogel beads was investigated.

## 2. Results and Discussion

### 2.1. Structural Characterization

**Scheme 1:**
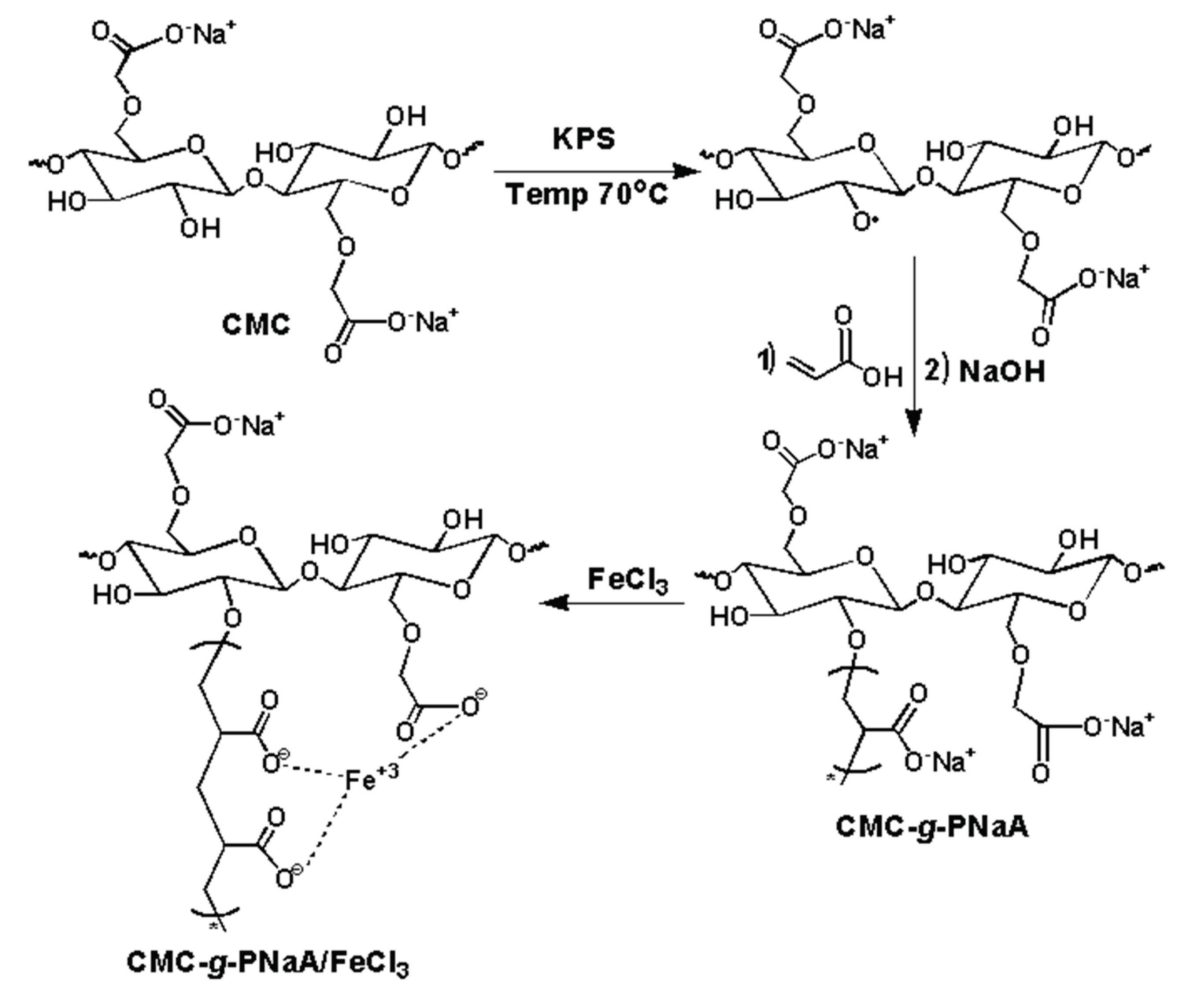
Schematic presentation of ionic cross-linked CMC-*g*-PNaA/FeCl_3_.

**Figure 1:**
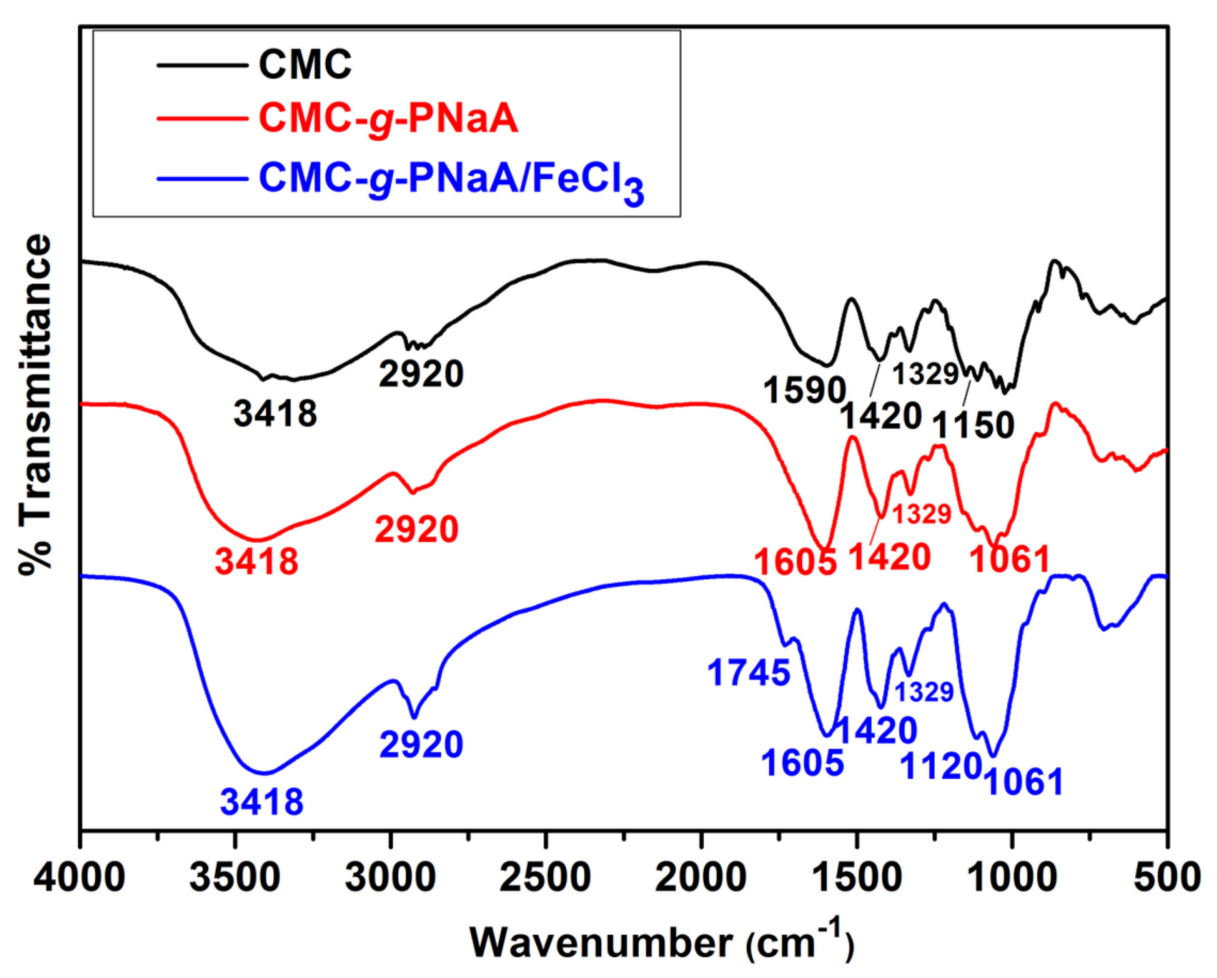
Fourier transform infrared spectroscopy (FTIR) of CMC, CMC-*g*-PNaA and CMC-*g*-PNaA/FeCl_3_.

Ionic cross-linked CMC-*g*-PNaA/FeCl_3_ hydrogel beads were prepared by the cross-linking of CMC-*g*-PNaA, followed by the grafting polymerization of AA onto CMC, as shown in Scheme 1. The homopolymer could be formed during the polymerization, but it may be mostly dissolved during the precipitation of reaction mixture. Cross-linked CMC-*g*-PNaA/FeCl_3_ hydrogel beads were formed by the intra-molecular interaction of ionic metals with CMC-*g*-PNaA chains. Nevertheless, inter-molecular cross-linking might be induced by the interaction of ionic metals with CMC-*g*-PNaA and the remaining content of the homopolymer [30]. In Figure 1, as reported in literature [1,31,32,33], CMC depicts the Fourier transform infrared spectroscopy (FTIR) absorption band at 1590 cm^−1^ and 1420 cm^−1^ ascribed to the carboxylate groups, which are a characteristic band from CMC (Figure 1). The broad absorption bands at around 3418 cm^−1^ are attributed to the –OH stretching, while the band 2920 cm^−1^ corresponds to C–H stretching. Furthermore, the bands between 1000–1200 cm^−1^ are attributed to the C–O stretching [34]. In the FTIR of CMC-*g*-PNaA, the band intensities of C-H stretching at 2920 cm^−1^ and carboxylate group at 1590 cm^−1^ and 1420 cm^−1^ were increased after the grafting of neutralized AA onto CMC [35], which confirms the successful formation of CMC-*g*-PNaA. Furthermore, in case of CMC-*g*-PNaA/FeCl_3_, a new band at 1745 cm^−1^ appeared due to the electrostatic interaction between CMC-*g*-PNaA and Fe^3+^ ions [22]. This new band confirms the formation of ionic cross-linked CMC-*g*-PNaA/FeCl_3_. Moreover, all absorption bands of CMC (3418, 2920, 1590 1420, 1329 and 1200–1000 cm^−1^) and CMC-*g*-PNaA (3418, 2920, 1605, 1420, 1329 and 1061 cm^−1^) are visible in the ionic cross-linked CMC-*g*-PNaA/FeCl_3_. The absorption bands of CMC in the spectrum of CMC-*g*-PNaA/FeCl_3_ are similar with the cellulose skeleton.

**Figure 2:**
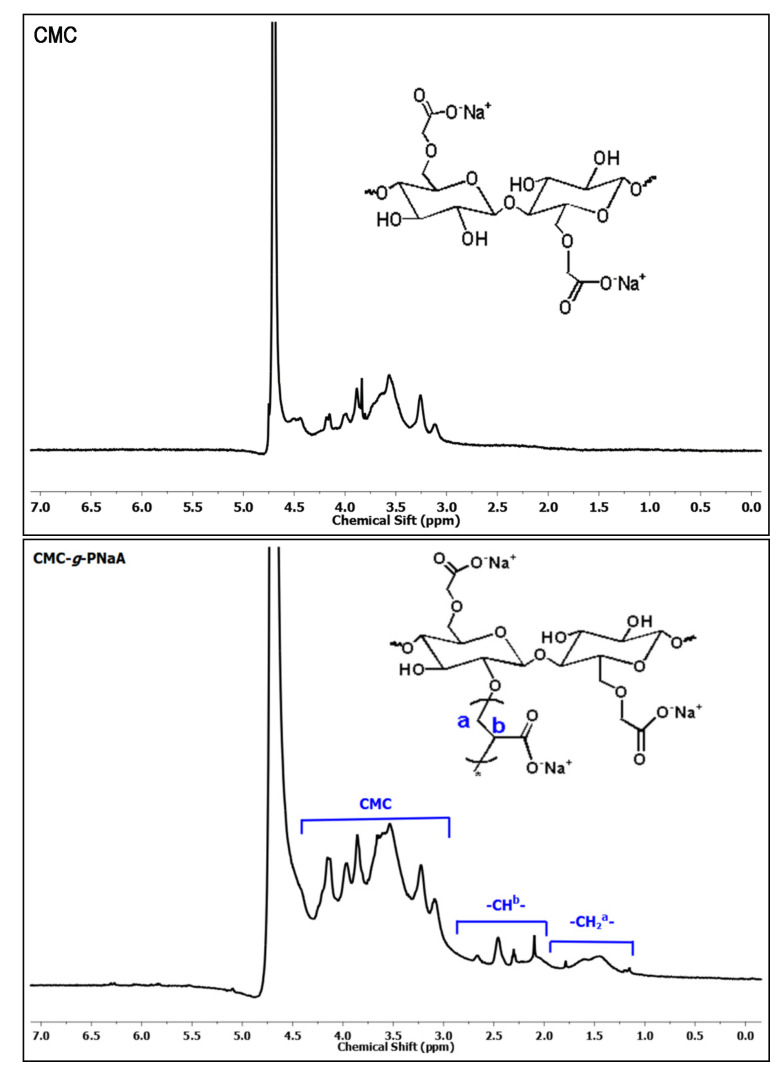
^1^H nuclear magnetic resonance (NMR) spectrum of CMC and CMC-*g*-PNaA.

**Figure 3:**
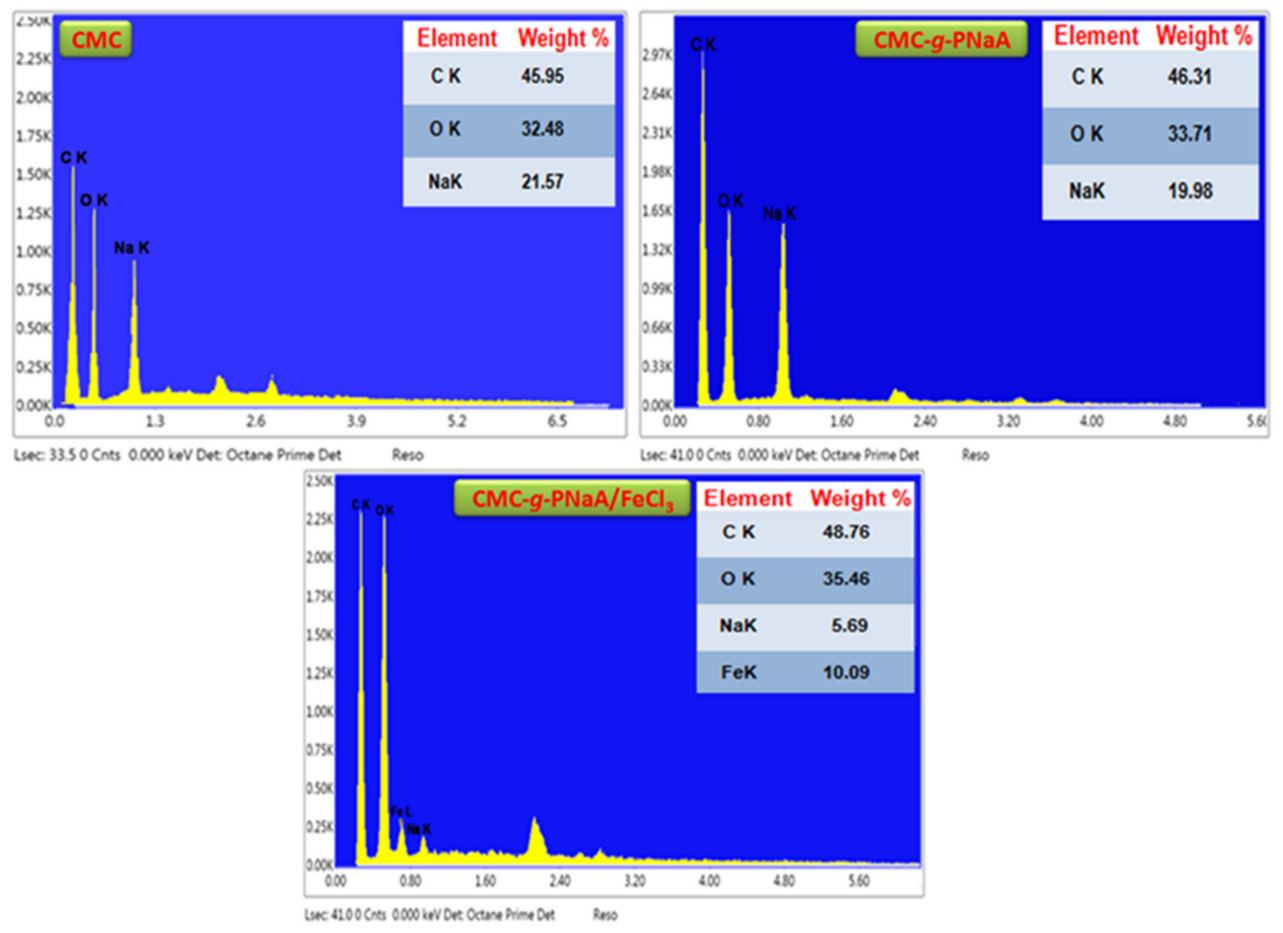
Energy dispersive X-ray analysis (EDX) spectra of CMC, CMC-*g*-PNaA and CMC-*g*-PNaA/FeCl_3_ hydrogel beads.

Furthermore, nuclear magnetic resonance (NMR) spectroscopy was employed to characterize the CMC and CMC-*g*-PNaA. CMC showed characteristic signals in the region of 3.0 ppm to 4.3 ppm (Figure 2). Moreover, the new characteristic signals appeared in the NMR spectra of CMC-*g*-PNaA in the regions of 1.11–1.93 ppm and 1.97–2.88 ppm, which were obtained due to the –CH_2_^a^- and –CH^b^- of PAA content (Figure 2) [35,36] and confirms the successful formation of CMC-*g*-PNaA. In addition, the successful grafting of CMC-*g*-PNaA copolymer and its effective ionic-crosslinking with Fe^3+^ ions was confirmed by energy dispersive X-ray analysis (EDX) spectra, as shown in Figure 3. CMC-*g*-PNaA exhibited increased content of carbon and oxygen elements than that of only CMC. Here, the peak intensity of the C and O elements was increased, which demonstrated a successful grafting of CMC and PNaA. Furthermore, in EDX spectra of CMC-*g*-PNaA/FeCl_3_, a new peak of the Fe element emerged, and the peak intensity of the Na element was significantly decreased after the ionic-crosslinking of CMC-*g*-PNaA with Fe^3+^ ions. This indicates the effective ionic-crosslinking between Fe^3+^ ions and polymeric chains (see Scheme 1).

**Figure 4:**
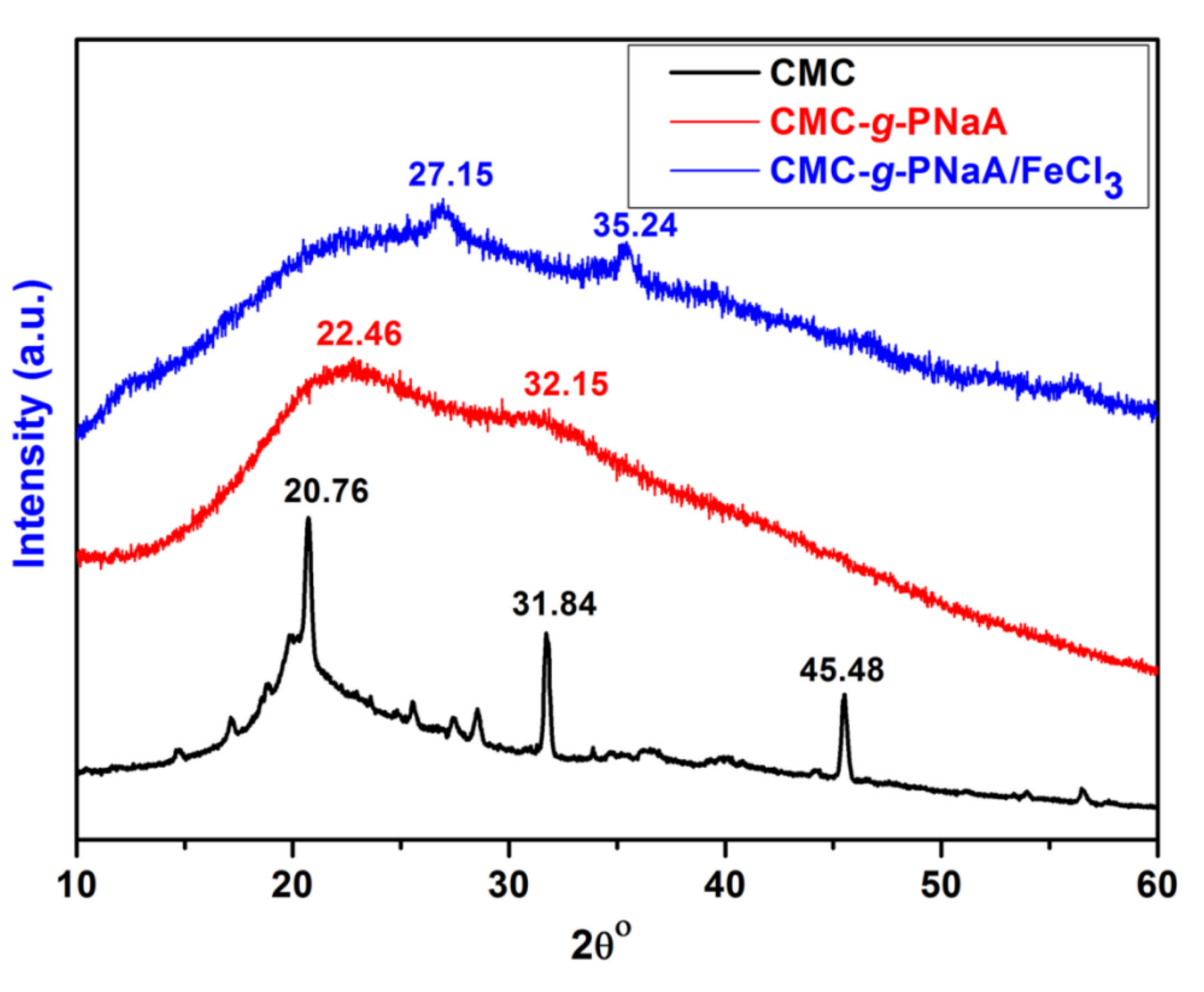
X-ray diffraction (XRD) patterns of CMC, CMC-*g*-PNaA and CMC-*g*-PNaA/FeCl_3_ hydrogel beads.

X-ray diffraction (XRD) patterns of CMC show the characteristic peaks at 2θ = 20.76°, 31.84° and 45.48°, which indicate the amorphous and crystalline structure. Furthermore, CMC-*g*-PNaA shows broad peaks at around 22.46° and 32.15°, which demonstrate that the crystalline region of CMC is destroyed after grafting of AA onto CMC. Meanwhile, CMC-*g*-PNaA/FeCl_3_ depicts a broad peak with two small intense peaks at around 27.15° and 35.24°, due to the ionic cross-linking of CMC-*g*-PNaA with Fe^3+^ ions, as shown in Figure 4 [37]. After ionic cross-linking, the XRD peaks of CMC-*g*-PNaA were shifted from the lower to higher 2θ values, which indicate the successful ionic cross-linking of Fe^3+^ with CMC-*g*-PNaA chains.

### 2.2. Thermal Gravimetric Analysis (TGA)

**Figure 5:**
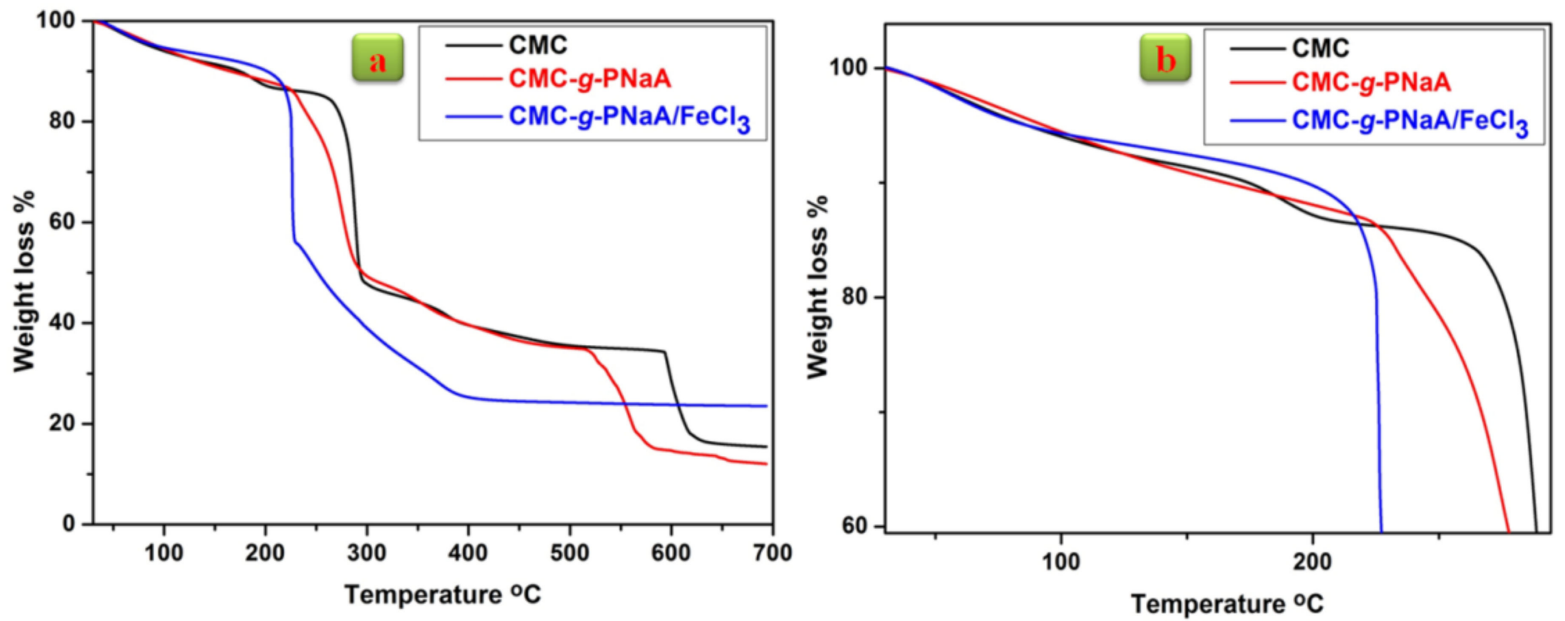
(**a**) Thermogravimetric analysis (TGA) curves of CMC, CMC-*g*-PNaA CMC-*g*-PNaA/FeCl_3_; and their corresponding (**b**) extended TGA curves.

Compared to the CMC and CMC-*g*-PNaA, CMC-*g*-PNaA/FeCl_3_ exhibits the one step degradation (Figure 5). In CMC, the major decomposition started at 240 °C, with 14.34% weight loss due to inorganic moiety, including minor weight (4.61%) loss from 154 °C to 202 °C. Moreover, the decomposition after 335 °C occurred due to the pyrolysis reaction [1]. Below 120 °C, the minor weight loss in all samples occurred due to the loss of moisture. CMC-*g*-PNaA shows the continued degradation up to 189 °C with 11.47% weight loss, which may be attributed to the desorbed water. Afterwards, the major degradation occurred from 189 °C to 400 °C with 48.94% weight loss, which may be attributed to the degradation of the side group and chain scission. The last degradation appeared after 400 °C with 27.35% weight loss, due to the pyrolysis reaction and chain scission [35]. Furthermore, CMC-*g*-PNaA/FeCl_3_ demonstrated initial degradation at 199 °C with 9.6% weight loss due to the moisture. After that, the main degradation was attributed up to 472 °C with 66.37% weight loss, which may be occurred due to side group scission, side chain scission and pyrolysis degradation of the CMC unit. Moreover, CMC-*g*-PNaA/FeCl_3_ reveals the higher residue of around 23% than those of CMC (15%) and CMC-*g*-PNaA (12%), as shown in (Figure 5a). Thermogravimetric analysis (TGA) results demonstrate that CMC-*g*-PNaA/FeCl_3_ exhibits higher thermal stability of 199 °C. The thermal stability of CMC-*g*-PNaA/FeCl_3_ is comparable with the previously reported literature of grafted-based polysaccharides hydrogel [38,39].

### 2.3. Morphological Analysis

**Figure 6:**
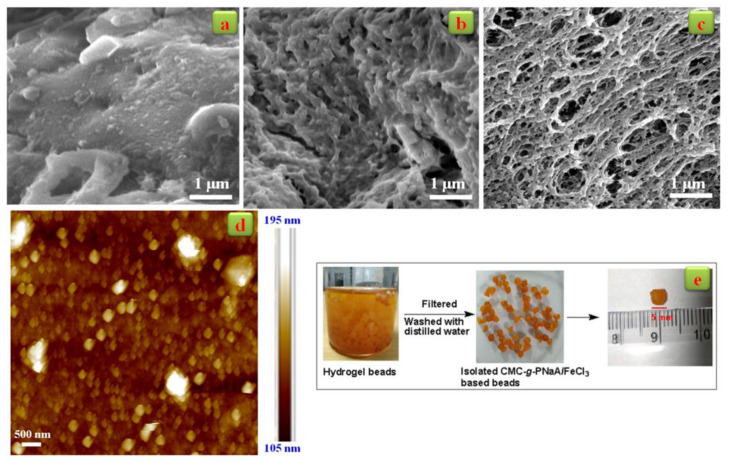
Scanning electron microscopy (SEM) images of (**a**) CMC; (**b**) CMC-*g*-PNaA; and (**c**) CMC-*g*-PNaA/FeCl_3_; (**d**) atomic force microscopy (AFM) image of CMC-*g*-PNaA/FeCl_3_ hydrogel bead; and (**e**) isolated beads of CMC-*g*-PNaA/FeCl_3_.

The surface morphology of CMC, CMC-*g*-PNaA and CMC-*g*-PNaA/FeCl_3_ hydrogel beads was analyzed by scanning electron microscopy (SEM) (Figure 6). CMC shows the crystalline and fiber type morphology (Figure 6a), while CMC-*g*-PNaA exhibits a porous morphology. The SEM results demonstrate that when AA was grafted onto CMC, the morphology was converted to porous with a different size and shape from crystalline and fiber-type morphology (Figure 6b). Moreover, CMC-*g*-PNaA/FeCl_3_ hydrogel beads depict the dense irregular-sized porous morphology as compared to the CMC-*g*-PNaA. Here, CMC-*g*-PNaA/FeCl_3_ showed the minimum and maximum pore size as around 66 nm and 800 nm, as were measured by ImageJ Software. Despite the large size of some pores (around 800 nm), the average pore size of CMC*-g*-PNaA/FeCl_3_ hydrogel beads was obtained at around 280 nm (Figure 6c). In addition, Figure 6d shows the atomic force microscopy (AFM) image of CMC-*g*-PNaA/FeCl_3_ hydrogel beads, and it indicates the granular topology of the obtained hydrogel beads (Figure 6e). Therefore, AFM analysis depicts the homogenous granular morphology of CMC-*g*-PNaA/FeCl_3_ hydrogel beads. The average surface roughness (R_a_) and root mean square roughness (R_ms_) of beads were obtained to be 10.8 nm and 107 nm, respectively.

### 2.4. Swelling Study

**Figure 7:**
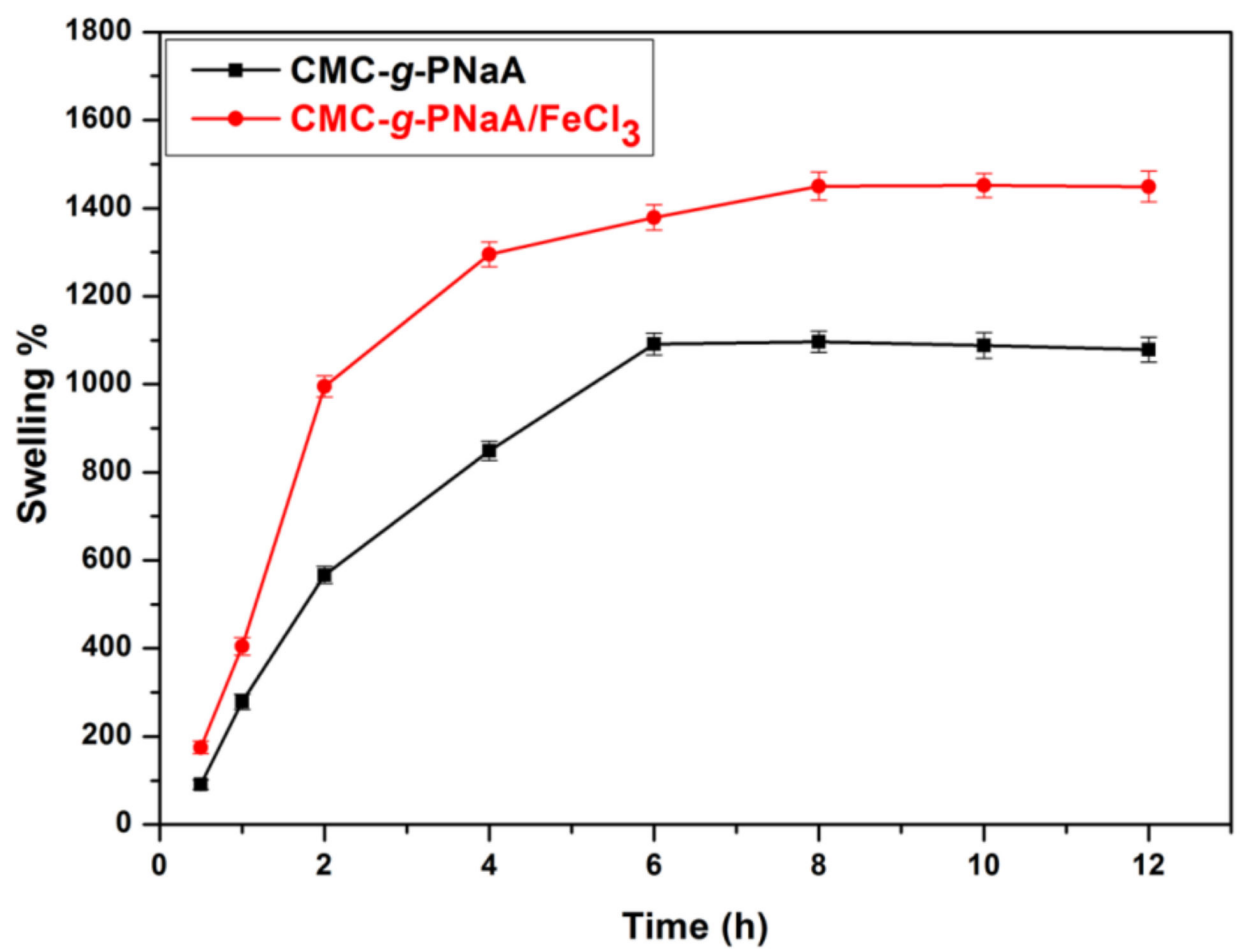
Swelling behavior of CMC-*g*-PNaA and CMC-*g*-PNaA/FeCl_3_ hydrogel beads.

The swelling behavior of CMC-*g*-PNaA and CMC-*g*-PNaA/FeCl_3_ hydrogel beads is shown in Figure 7 as a function of time. It was found that the swelling rate of ionic cross-linked CMC-*g*-PNaA/FeCl_3_ hydrogel beads is initially very high, but after 8 h, the hydrogel beads reached the equilibrium swelling rate. CMC-*g*-PNaA/FeCl_3_ hydrogel beads showed the highest swelling rate of up to 1452% in the time range of 10 h to 12 h. In addition, the swelling rate of CMC-*g*-PNaA increased gradually with time up to 6 h, while it started to decrease after 10 h due to the breaking of the polymeric chain. CMC-*g*-PNaA exhibits the equilibrium swelling rate as around 1096% [38]. Moreover, the higher swelling percentage of CMC-*g*-PNaA/FeCl_3_ may be attributed to the higher porosity of hydrogel beads. The higher swelling rate of CMC-*g*-PNaA/FeCl_3_ exhibits as compared to CMC-*g*-PNaA may be attributed to the ionic cross-linking, resulting in a higher number of pores with a greater pore size as compared to the CMC-*g*-PNaA.

## 3. Conclusions

In this study, novel CMC-*g*-PNaA/FeCl_3_ hydrogel beads were prepared based on ionic-interactions between CMC-*g*-PNaA and FeCl_3_ as an ionic-crosslinker. TGA demonstrated that the CMC-*g*-PNaA/FeCl_3_ hydrogel beads showed higher thermal stability at around 199 °C as compared to CMC-*g*-PNaA, which was stable up to 189 °C. CMC-*g*-PNaA/FeCl_3_ hydrogel beads showed higher pore density with an average pore size of 280 nm. Furthermore, AFM images revealed the granule type topology of beads with R_a_ = 10.8 nm. CMC-*g*-PNaA/FeCl_3_ hydrogel beads showed a swelling percentage of 1452% as compared to 1096% for CMC-*g*-PNaA. Overall, the ionic linkage between CMC-*g*-PNaA and FeCl_3_ significantly improved the physical properties of the hydrogel, and the obtained CMC-*g*-PNaA/FeCl_3_ hydrogel beads could have potential applications in waste water treatment and agriculture.

## 4. Materials and Methods

### 4.1. Materials

CMC (low viscosity; 50–200 cP) was procured from Sigma Aldrich (Bangalore, India). AA ≥ 99%, FeCl_3_ ≥ 98%, Sodium hydroxide (NaOH, ≥98%) and Acetone and Potassium persulfate (KPS, ≥99%) were procured from Hi-Media (Mumbai, India). All the materials were used as received, without any purification.

### 4.2. Synthesis of CMC-*g*-PNaA

CMC-*g*-PNaA was synthesized by the following procedure [38,40]: firstly, 1.0 g of CMC was dissolved in 50 mL distilled water for 1 h at room temperature, and an inert atmosphere in a three neck flask equipped with a magnetic stirrer. Following this, KPS (0.2 g) initiator was added into the CMC solution at 70 °C, and after 30 min, neutralized AA (3.5 mL) was inserted into the reaction mixture and heated for 2 h. Thereafter, the pH of the reaction mixture was raised up to 8 by using a NaOH solution, and the mixture was precipitated by acetone. Finally, CMC-*g*-PNaA precipitate was collected and dried in a vacuum oven for 24 h at 60 °C.

### 4.3. Preparation of CMC-*g*-PNaA/FeCl_3_ Hydrogel Beads

CMC-*g*-PNaA/FeCl_3_ hydrogel beads were prepared by the following procedure [22]: firstly, CMC-*g*-PNaA (2% *w/v*) was dissolved in distilled water by using a mechanical stirrer at 300 rpm for 6 h. Then, 50 mL of CMC-*g*-PNaA solution was added dropwise in the 100 mL aqueous solution of FeCl_3_ (0.020 mol). After that, the obtained spherical hydrogel beads were filtered, washed with distilled water and dried in vacuum oven for 24 h.

### 4.4. Characterization

FT-IR spectra of the CMC, CMC-*g*-PNaA and CMC-*g*-PNaA/FeCl_3_ were recorded using FT-IR spectroscopy (Perkin Elmer FT-IR C91158 Spectrum, Waltham, MA, USA) within the range of 4000 cm^–1^ to 500 cm^–1^.

^1^H-NMR spectra of CMC and CMC-*g*-PNaA was obtained in D_2_O solvent with the help of a NMR spectrometer (Bruker 500 MHz, Rheinstetten, Germany).

The elemental analysis of CMC, CMC-*g*-PNaA and CMC-*g*-PNaA/FeCl_3_ was analyzed by the EDX (Mira 3 Tescan, Kohoutovice, Czech Republic) instrument.

XRD spectra were obtained using a Rigaku Ultima IV diffractometer (Tokyo, Japan) equipped with a Cu-Kα radiation (λ = 0.154 nm) in the 2θ range of 5°–60°, with a voltage of 40 kV and a current of 40 mA.

The thermal stability of the CMC, CMC-*g*-PNaA and CMC-*g*-PNaA/FeCl_3_ was analyzed by TGA using Exstar TGA/DTG 6300 (Tokyo, Japan) at a 10 °C min^–1^ heating rate in the presence of nitrogen (flow rate: 200 mL/min).

The hydrogel surface morphology and pore size of CMC-*g*-PNaA/FeCl_3_, along with CMC and CMC-*g*-PNaA, was evaluated by a SEM (Mira 3 Tescan, Kohoutovice, Czech Republic) instrument. Topographical analysis of CMC-*g*-PNaA/FeCl_3_ was performed by Bruker Dimension Icon Atomic Force Microscope, USA.

The swelling % of CMC-*g*-PNaA and CMC-*g*-PNaA/FeCl_3_ hydrogel beads was performed at room temperature with the help of tea-bag methods as a function of time. For this, dried hydrogel beads (0.02 g) were added to clean empty tea-bags and immersed in 100 mL water. Furthermore, swollen hydrogel beads were taken out at regular time intervals, and after that the weight was measured. The swelling behavior (%) was determined as follows [41]:

$$\mathrm{Swelling}\;\%=\frac{\left(W_{2}-W_{1}\right)}{W_{1}}\;\times \;100$$

where *W*_1_ is the weight of the dried hydrogel bead, while the *W*_2_ is the weight of the swollen hydrogel bead.
